# Apoptosis Is Essential for Neutrophil Functional Shutdown and Determines Tissue Damage in Experimental Pneumococcal Meningitis

**DOI:** 10.1371/journal.ppat.1000461

**Published:** 2009-05-29

**Authors:** Uwe Koedel, Tobias Frankenberg, Susanne Kirschnek, Bianca Obermaier, Hans Häcker, Robert Paul, Georg Häcker

**Affiliations:** 1 Department of Neurology, Clinic of the University of Munich, Munich, Germany; 2 Institute for Medical Microbiology, Technische Universität München, Munich, Germany; 3 Department of Infectious Diseases, St. Jude Children's Research Hospital, Memphis, Tennessee, United States of America; University of Pennsylvania, United States of America

## Abstract

During acute bacterial infections such as meningitis, neutrophils enter the tissue where they combat the infection before they undergo apoptosis and are taken up by macrophages. Neutrophils show pro-inflammatory activity and may contribute to tissue damage. In pneumococcal meningitis, neuronal damage despite adequate chemotherapy is a frequent clinical finding. This damage may be due to excessive neutrophil activity. We here show that transgenic expression of Bcl-2 in haematopoietic cells blocks the resolution of inflammation following antibiotic therapy in a mouse model of pneumococcal meningitis. The persistence of neutrophil brain infiltrates was accompanied by high levels of IL-1β and G-CSF as well as reduced levels of anti-inflammatory TGF-β. Significantly, Bcl-2-transgenic mice developed more severe disease that was dependent on neutrophils, characterized by pronounced vasogenic edema, vasculitis, brain haemorrhages and higher clinical scores. *In vitro* analysis of neutrophils demonstrated that apoptosis inhibition completely preserves neutrophil effector function and prevents internalization by macrophages. The inhibitor of cyclin-dependent kinases, roscovitine induced apoptosis in neutrophils *in vitro* and *in vivo*. In wild type mice treated with antibiotics, roscovitine significantly improved the resolution of the inflammation after pneumococcal infection and accelerated recovery. These results indicate that apoptosis is essential to turn off activated neutrophils and show that inflammatory activity and disease severity in a pyogenic infection can be modulated by targeting the apoptotic pathway in neutrophils.

## Author Summary

Infections are typically accompanied by inflammation and the presence of leucocytes at the infected site. Especially in pyogenic (pus generating) infections, neutrophils are the main leukocytes that arrive and that combat the infection. When the bacteria have been killed, the inflammation also has to be resolved, and this resolution involves the death of the neutrophils through the process of apoptosis. Although we know that neutrophils eventually die through apoptosis, the connection between neutrophil apoptosis and inflammation in bacterial infections has not been clear. In meningitis, bacteria gain access to the space surrounding the brain, multiply and cause inflammation. Despite antibiotic therapy, human pneumococcal meningitis often causes brain damage and death of the patient. We show here that the experimental inhibition of neutrophil apoptosis leads to much more severe clinical disease in a mouse model of bacterial (pneumococcal) meningitis. This was due to a prolonged inflammatory activity of the neutrophils. Experimental induction of neutrophil apoptosis in mice that had been infected and treated with antibiotics (mimicking human meningitis) improved the clinical condition and accelerated recovery. Experimental manipulation of neutrophil apoptosis can therefore be beneficial in acute inflammation.

## Introduction

The coordinated termination of immune responses is critical to prevent autoimmunity and immunopathology, and the varying effector systems of the immune response are likely to require different ways for their termination. Against bacterial and fungal infections, the first line of defence is formed by neutrophils. In acute infections, there is typically a rapid influx of neutrophils from the blood into the site of infection, which is often followed by the infiltration of monocytes that then differentiate into inflammatory macrophages [1].

Neutrophil tissue invasion can be triggered by infection or injury and is mediated especially by lipid chemoattractants such as leukotrienes (for a recent review see [2]). The removal of neutrophils in the resolution phase occurs through neutrophil apoptosis and phagocytic uptake by inflammatory macrophages [3]. As neutrophils contain or can generate a number of toxic substances and intermediates, it is considered critical that they die by apoptosis, since in the course of apoptotic cell death the plasma membrane stays intact until the dying cell has been ingested by a macrophage [3],[4].

Severe infections with pyogenic bacteria cause a massive influx of neutrophils and strong signs of inflammation and can lead to permanent tissue damage at the site of infection. Human pneumococcal meningitis is a frequent and problematic infectious disease, not only because of its high lethality but also because patients, despite rapid and optimal antibiotic treatment often are left with neurological deficits as a consequence of brain tissue damage [5]. It has been speculated in the past, and circumstantial evidence has been provided for this [6],[7], that the neutrophil-dominated inflammatory response contributes to this outcome in pneumococcal meningitis.

Neutrophils in circulation are extremely short-lived before they undergo apoptosis and are taken up by macrophages [8]. A number of interleukins and other signals have been identified that can extend the lifespan of neutrophils and that probably operate to keep them alive in the inflammatory environment for long enough to be effective at combating bacteria [9],[10]. In a model of non-infectious, carrageenan-induced pleurisy in rats, experimental enhancement of apoptosis in neutrophils was able to reduce the number of inflammatory cells while a peptide that delayed neutrophil apoptosis was able to enhance inflammatory cell number [11] indicating that in sterile inflammation apoptosis regulates the number of inflammatory cells. A recent report further demonstrates that experimental induction of apoptosis in models of non-infectious inflammation can enhance resolution of the process [12]. These results suggest that neutrophil presence indeed can regulate inflammation in these cases and indicate the possibility that the level of apoptosis may also determine the clinical outcome of pyogenic infections upon antibiotic treatment (i. e. when the infectious agent has been cleared away therapeutically).

Neutrophils are said to ‘age’ before undergoing programmed cell death. It is not clear, however, whether ageing is the expression of an independent molecular program in neutrophils or is simply the state close to physiological apoptosis. Mouse neutrophils over-expressing transgenic Bcl-2 or lacking the pro-apoptotic Bcl-2 protein Bim show strongly reduced spontaneous apoptosis [13],[14]. These results indicate that the activation of Bim initiates spontaneous neutrophil apoptosis while high-level expression of Bcl-2 can block this process.

We here test how neutrophil apoptosis affects inflammation and disease severity in experimental meningitis. When neutrophils were kept alive by Bcl-2 in transgenic mice, the inflammation was exacerbated, which indicates that neutrophils that were kept experimentally alive were able to maintain their pro-inflammatory activity. This was corroborated by *in vitro* analyses that show that such ‘undead’ cells are indeed fully competent functionally. We finally used the drug roscovitine to induce apoptosis in wt neutrophils *in vivo*. This treatment substantially improved recovery of antibiotics-treated mice. In the resolution of a pyogenic infection, inflammation (and tissue damage) is thus determined by the lifespan of neutrophils, and therapeutic targeting of the apoptotic pathway can modulate the clinical outcome.

## Results

### Inhibition of apoptosis by Bcl-2 maintains inflammation *in vivo* in experimental meningitis

Bacterial, especially pyogenic infections are dominated by the influx of neutrophils which, at the end of the infection, die and are taken up by macrophages. A reduction in physiological apoptosis might result in prolonged neutrophil presence at the site of inflammation. Because of their capacity for the production of toxic mediators, this continued neutrophil presence could result in hyperinflammatory tissue damage. We tested this hypothesis in a model of mouse pneumococcal meningitis. In this model, pneumococci are directly inoculated into the cerebrospinal fluid of mice via puncture of the cisterna magna. This inoculation causes the rapid influx of neutrophils from blood, leading to pathological changes that closely mimic those of human pneumococcal meningitis [7],[15]. Despite this neutrophil recruitment, the host defence inside the CNS is unable to eradicate the pathogen, without antibiotic therapy meningitis almost invariably causes the death of the animals [16] (as is the case in human meningitis [17]). To rescue the mice and to study the resolution phase of the inflammation, mice have to be treated with antibiotics (beginning in this model usually at 24 h post infection). This treatment results in near-complete killing of the bacteria as very few bacteria can be cultured from CSF or brain tissue within 24 h after application of antibiotics [18]. Following application of the antibiotic, the inflammation begins to resolve and apoptosis of neutrophils can be readily observed *in situ* and this apoptosis peaks at 24 h after initiation of treatment (i. e. at 48 h post-infection) [15]. We used this mouse model to analyse the contribution of apoptosis to the resolution of inflammation. Wt mice were compared to *vav-bcl-2* transgenic mice. These mice express human Bcl-2 in all haematopoietic lineages including neutrophils [19]. Neutrophils isolated from the bone marrow of these mice have been shown to be protected from spontaneous apoptosis *in vitro* [13].

**Figure 1:**
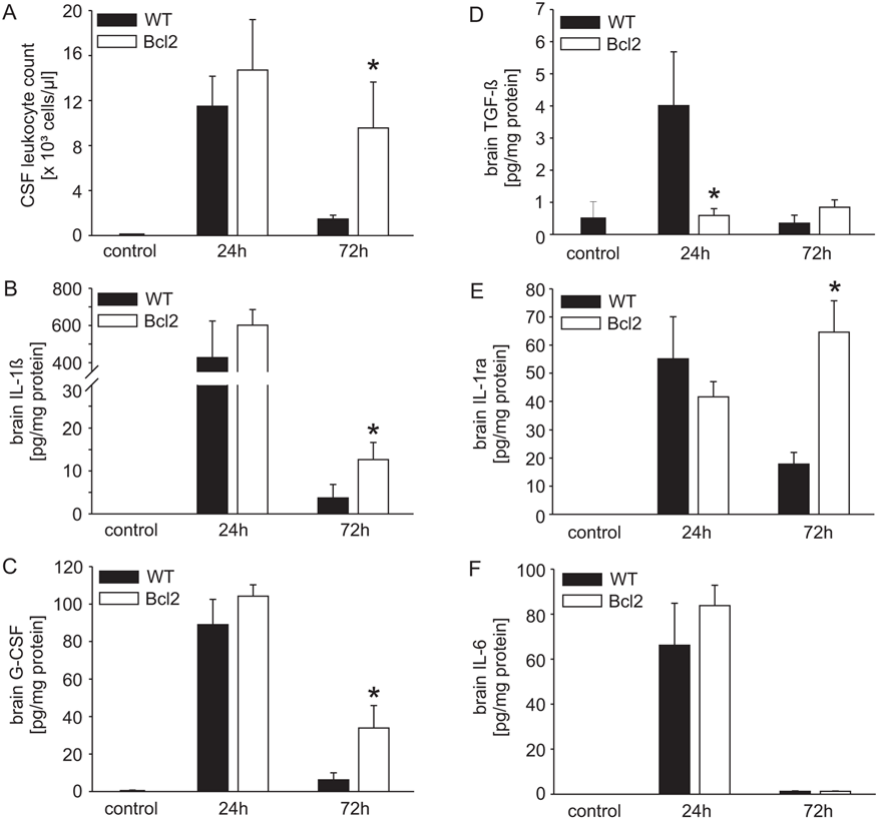
Transgenic Bcl-2 blocks the resolution of inflammation in pneumococcal meningitis. Groups of 7 mice (24 h) and 12 mice (72 h) were infected with pneumococci and in the case of 72 h analysis treated with antibiotics starting at 24 h. (A) CSF leucocyte counts were determined in samples obtained by puncture of the cisterna magna by counting in a Fuchs-Rosenthal chamber. Absolute neutrophil counts were calculated on the basis of these numbers and the relative numbers of neutrophils as assessed by Giemsa staining of CSF smears. These numbers were 10,122±5,763 (mean/SD of 6 mice) in wt and 11,471±6,036 in Bcl-2 transgenic mice (not significantly different). (B–F) cytokine levels were determined in brain lysates and are expressed as levels per total protein. (B) Levels of IL-1β, (C) levels of G-CSF; *P<0.05, Bcl-2 transgenic mice compared to wt mice at 72 h. (D) Levels of active TGF-ß in brain homogenates of wt mice (n = 7 (0 h), 7 (24 h), 12 (48 h), 12 (72 h)) and Bcl-2 transgenic mice (n = 7 (24 h) and n = 9 (72 h)), *P<0.05, (wt mice vs. Bcl-2 transgenic mice). (E) Levels of IL-1 receptor antagonist (IL-1ra; *P<0.05, Bcl-2 transgenic mice compared to wt mice at 72 h), (F) levels of IL-6.

Upon intracisternal injection, both wt and Bcl-2 transgenic mice developed fulminant disease. At 24 h after inoculation, all infected mice presented clinical signs of severe illness such as reduced vigilance, impaired motor function, weight loss and hypothermia, irrespective of their genotype. No differences were seen at 24 h between the two mouse strains with regard to meningitis-associated intracranial complications (e.g. blood-brain barrier breakdown, see below), CNS inflammation (as indicated by cellular infiltrate and high levels of IL-1β and G-CSF, Fig. 1A–C) and bacterial outgrowth from the CNS (6.4+/−0.3 log cfu/ml cerebellar lysate in wt mice vs. 6.4+/−0.2 log cfu/ml in Bcl-2 transgenic mice). In pneumococcal meningitis, neutrophils are unable to control bacterial replication and bacterial titres in the CNS are therefore independent of neutrophil numbers, even when neutrophils are depleted (Fig. S2D). There was no difference in CSF leucocyte and neutrophil numbers between the mouse strains (Fig. 1A; absolute numbers of neutrophils were 10,122±5,763 (mean/SD of 6 mice) in wt and 11,471±6,036 in Bcl-2 transgenic mice) prior to antibiotic treatment. Following treatment there was a massive drop in CSF leukocyte counts in wt mice but this drop was strongly reduced in Bcl-2-transgenic mice, resulting in about 5 times more leukocytes in the CSF compared to wt mice at 72 h after infection (Fig. 1A). This indicates that apoptosis is essential for the reduction in CSF leukocyte counts as observed upon successful antibiotic therapy; if apoptosis is inhibited the CSF leukocyte numbers will stay up (percentages of apoptotic leukocytes in wt vs. Bcl-2 transgenic mice at 24 h after initiation of antibiotic therapy – when started at 18 hours after infection were: 13.7+/−1.5% vs. 3.7+/−1.5%). Reduced neutrophil apoptosis thus leads to a prolonged presence of neutrophils at the site of inflammation in this model of meningitis.

To assess whether this prolonged presence of neutrophils affected the course of inflammation and disease, we measured disease severity and parameters of tissue damage. Significantly increased levels of IL-1β, IL-1 receptor antagonist (IL-1ra) and G-CSF but not IL-6 were detected in the brains of Bcl-2-transgenic mice as compared to wt mice at 72 h (about 4 times as high, Fig. 1B,C,E,F), suggesting that neutrophils were not only present but also continued with their pro-inflammatory functions. We have previously shown that the loss of caspase-1 (which is required for the release of mature IL-1β) in mice alleviates inflammation [20] and this prolonged production of this cytokine may therefore contribute to the hyperinflammatory phenotype. Levels of IL-1 receptor antagonist also remained high in Bcl-2 transgenic animals as compared to wt animals while levels of IL-6 dropped in both strains of mice (Fig. 1E,F). These data are indicative of a prolonged pro-inflammatory activity in the brain of Bcl-2 transgenic mice.

Apoptotic neutrophils are taken up by macrophages, and the uptake of apoptotic cells by macrophages has been suggested to have anti-inflammatory effects. The two anti-inflammatory cytokines TGF-β and IL-10 have been found to be secreted by macrophages that have taken up apoptotic cells and are thought to be mediators of the anti-inflammation. The persistence of the infiltrate in Bcl-2 transgenic mice following meningitis *in vivo* suggested that neutrophils were not taken up by macrophages, and this might result in reduced secretion of TGF-β (IL-10 is not detectable in this model [15]). We therefore measured the levels of TGF-β in brain homogenate from infected mice. In wt mice, the levels of TGF-β reached a peak at about 24 h and started to decline after 48 h; baseline was reached again at about 72 h (Fig. 1D). In brains from Bcl-2 transgenic mice, this increase in TGF-β levels was not observed; the concentrations were close to baseline levels at both 24 h and 72 h (Fig. 1D). A reduced secretion of TGF-β, a likely consequence of reduced uptake of apoptotic neutrophils, may therefore contribute to the increased inflammation in Bcl-2 transgenic animals.

### Transgenic Bcl-2 leads to enhanced tissue damage and exacerbation of meningitis

**Figure 2:**
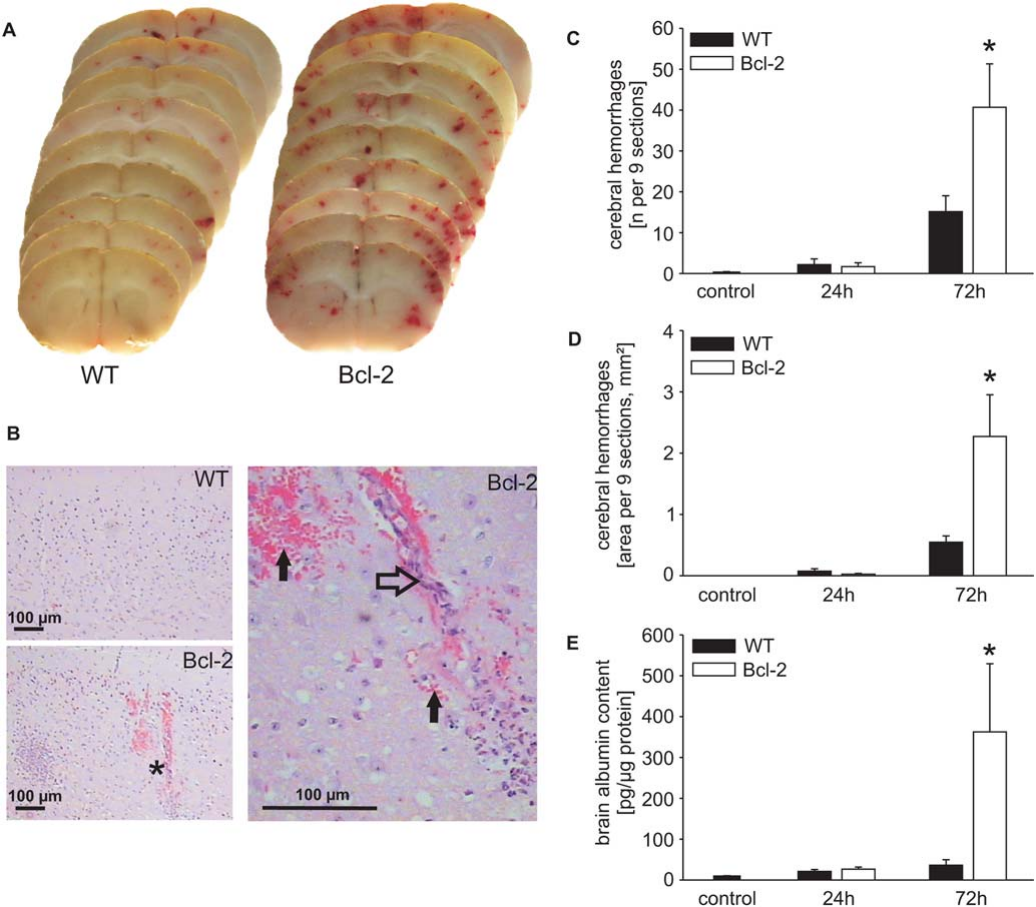
Exacerbation of meningitis-induced intracranial complications in Bcl-2 transgenic mice. Brain sections from wt or Bcl-2 transgenic mice at 72 h post infection were assessed macroscopically (A) or microscopically in H&E stained sections (B). Cortical haemorrhages are easily visible. (B) The area indicated by an asterisk in the bottom left image is shown at higher magnification on the right. Note the extensive vasculitis (open arrow) and extravasated erythrocytes (closed arrows) suggesting structural damage of the vessel. (C,D) Enumeration and size of the bleeding areas detected. (E) Severe damage to the integrity of the blood brain barrier was also reflected by increased albumin concentrations in brain tissue from Bcl2 mice. *P<0.05, Bcl-2 transgenic mice compared to wt mice at 72 h.

Strikingly, the continued presence and activity of neutrophils in Bcl-2 transgenic mice was associated with an aggravation of tissue damage and with more severe disease. The macroscopic hallmarks of this disease model are multifocal cortical hemorrhages, which are found histologically to result from widespread leukocytoclastic vasculitis, predominantly of small vessels in the brain cortex [21]. More extensive hemorrhages were evident in brain sections in Bcl-2 transgenic mice, both macroscopically (Fig. 2A) and in tissue sections (Fig. 2B). Quantitative analysis showed a clear increase in both number (Fig. 2C) and bleeding area (Fig. 2D) in brains of Bcl-2 transgenic mice. More severe damage to the integrity of the blood brain barrier was reflected by the albumin concentrations in the CNS, which were about 10-fold higher in Bcl-2 transgenic mice than in wt mice (Fig. 2E). The enhanced bleeding and breach of the blood brain barrier were accompanied by the accumulation of inflammatory cells in the CSF-filled ventricular space (Fig. S1A) and infiltrates of cells positive for the neutrophil marker Gr-1 in the subarachnoid space and around small blood vessels in the brain (Fig. S1B). Enumeration of cell populations in the CSF showed that the difference in cell number between wt and Bcl-2-transgenic mice were due to increased numbers in neutrophils (Fig. S2B, isotype controls), suggesting a role of these cells in tissue damage. Further, comparison of relative cell numbers 24 h after antibiotic treatment showed that the difference between wt and Bcl-2 transgenic animals was that more neutrophils and fewer apoptotic cells were present in the CSF of Bcl-2 transgenic animals (Fig. S2C). This strongly suggested that the difference between the mouse strains was that neutrophils survived better in Bcl-2 transgenic mice. To test this more directly, wt or Bcl-2 transgenic animals were depleted of neutrophils by injection of anti-Gr-1 antibody (Fig. S2A). When Bcl-2-transgenic mice depleted of neutrophils were infected with pneumococci, haemorrhages were massively reduced (Fig. S2E,F) indicating that the brain tissue damage indeed depended on neutrophils. Wt mice showed fewer and much reduced hemorrhages compared to Bcl2-transgenic mice and depletion of neutrophils in wt mice led to complete disappearance of cerebral hemorrhages (Fig. S2F).

The histological findings suggested more severe disease in Bcl-2 transgenic mice. Indeed, the clinical scores were significantly higher in these mice at 72 h than in wt mice (wt 5.3+/−2.1; Bcl-2 transgenic 10.5+/−4.6; 12 mice in each group, p<0.02). The outcome of this model can be adjusted by using different pneumococcal strains and by varying the time of antibiotic treatment. In the protocol we used here, the model has a near zero mortality in wt mice, and while all 12 wt mice survived in this series of experiments, 3 out of 12 Bcl-2 transgenic mice died (a summary of the survival of mice is given in Fig. S6). Blockade of apoptosis is thus sufficient to block resolution of the inflammation that is induced by pyogenic infection.

### Bcl-2 rescues neutrophil function *in vitro*

These results strongly suggested that neutrophils that were experimentally kept alive by Bcl-2 maintained their inflammatory function. This would have to mean that neutrophil ‘ageing’ does not operate as long as the cells are not undergoing apoptosis, i. e. that ageing was mechanistically identical to apoptosis. We next established an *in vitro* system to address the question whether apoptosis is indeed required to turn off a neutrophil and whether inhibition of apoptosis can preserve neutrophil function. To block apoptosis, we again chose the over-expression of Bcl-2. The use of primary neutrophils from mice can be difficult due to experimental variation and low yield. We therefore chose a recently described system that allows expansion of neutrophil progenitor cells and differentiation *in vitro*. Neutrophil progenitor cells are immortalised by a fusion protein of the estrogen receptor and the oncoprotein Hoxb8, allowing unlimited expansion of these progenitors in the presence of stem cell factor (SCF). Withdrawal of estrogen turns off Hoxb8, allowing the differentiation into mature neutrophils *in vitro* [22].

Progenitor lines were established from wild type (wt) and *vav-bcl-2*-transgenic mice. Both lines of progenitors grew equally well in the presence of estrogen. Withdrawal of estrogen caused differentiation with indistinguishable kinetics in both lines, as measured by the appearance of neutrophil morphology and the expression of the neutrophil marker Gr-1 in the course of several days (Fig. S3).

**Figure 3:**
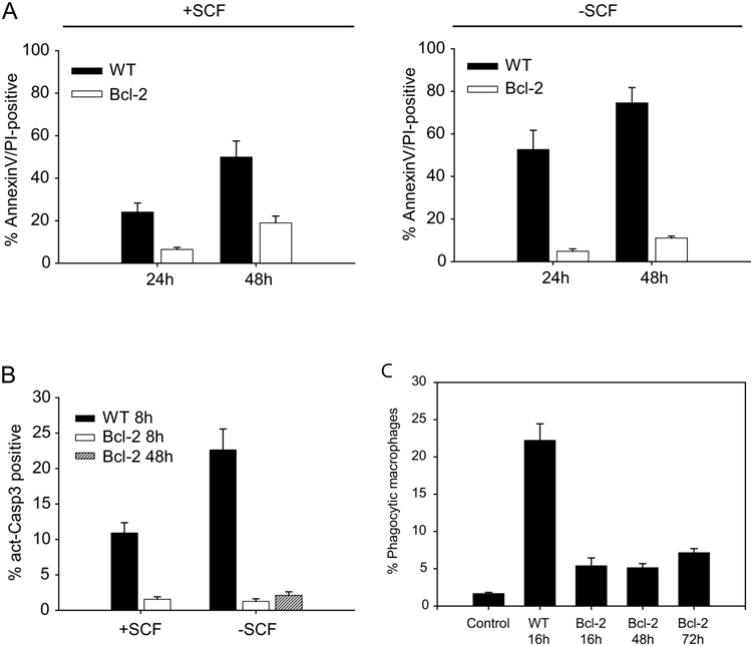
Overexpression of Bcl-2 protects neutrophils from apoptosis and removal by macrophages. (A) wt or Bcl-2-overexpressing neutrophils differentiated for 5 days were in addition cultured for 24 or 48 hours in the presence or absence of SCF. Cells were then stained with AnnexinV-FITC and propidium iodide (PI) and analysed by flow cytometry. Bars/error bars indicate mean/SEM of five independent experiments. (B) Neutrophils differentiated for 5 days were cultured for 8 or 48 hours in the presence or absence of SCF, followed by staining for active caspase-3. Error bars show the SEM of three independent experiments. (C) Neutrophils differentiated for 5 days were cultured in the absence of SCF for the indicated periods of time and stained with AnnexinV-PI or CFSE. Green-fluorescent (CFSE-stained) neutrophils were then added to cultures of RAW macrophages stained with the red dye PKH26 (ratio neutrophil∶macrophages, 5∶1). After 4 h of co-incubation, cultures were analysed by flow cytometric analysis. The proportion of phagocytic cells within the macrophage population is shown for wt or Bcl-2-expressing neutrophils at various time points. No double-positive cells were observed when reactions were carried out at 4°C (data not shown). Data are mean/SEM from 3 independent experiments.

We tested the anti-apoptotic effect of Bcl-2 against spontaneous neutrophil apoptosis in two conditions. Neutrophils were differentiated *in vitro* for five days in the presence of SCF. At day 5 to 6, wt neutrophils began to undergo spontaneous apoptosis (detected by staining for annexin V and propidium iodide and by nuclear morphology) despite the continued presence of SCF, reaching levels of about 60% apoptotic cells 48 h later (on day 7) (Fig. 3A; Fig. S4A gives an example of the primary data). When SCF was washed away on day 5, the apoptotic population was about 50% on day 6 and 80% on day 7. Under the same conditions, there was hardly any apoptosis detectable on day 6 in Bcl-2 neutrophils, while about 15–25% of cells were apoptotic on day 7. Surprisingly, there was no difference between Bcl-2 neutrophil cultures with and without SCF (Fig. 3A). This suggests that Bcl-2 was able completely to block apoptosis due to SCF-withdrawal but that there was a component of spontaneous apoptosis that was not inhibited by Bcl-2. Apoptosis was accompanied by rapid activation of caspase-3 in wt cells, and Bcl-2 blocked this activation (Fig. 3B and Fig. S4B).

Ageing neutrophils in circulation are phagocytosed and digested by spleen macrophages. To test whether apoptosis-inhibition by Bcl-2 also blocked ageing-associated uptake by macrophages, differentiated neutrophils were labelled with the fluorescent dye CFSE and incubated with cells from the mouse macrophage line RAW264.7. As shown in Fig. 3C and Fig. S4C, there was substantial uptake of wt neutrophils following 16 h of SCF-withdrawal (Fig. 2C; a large number of cells were apoptotic at this time, Fig. S4C). In Bcl-2 neutrophils, apoptosis was efficiently blocked even at 72 h of SCF-withdrawal, and there was very little uptake of the cells by RAW264.7 macrophages (Fig. S4C). Bcl-2 expression in RAW264.7 cells did not affect phagocytosis (data not shown). Bcl-2 therefore blocks not only apoptosis but also macrophage uptake of neutrophils that have gone beyond their physiological lifespan.

**Figure 4:**
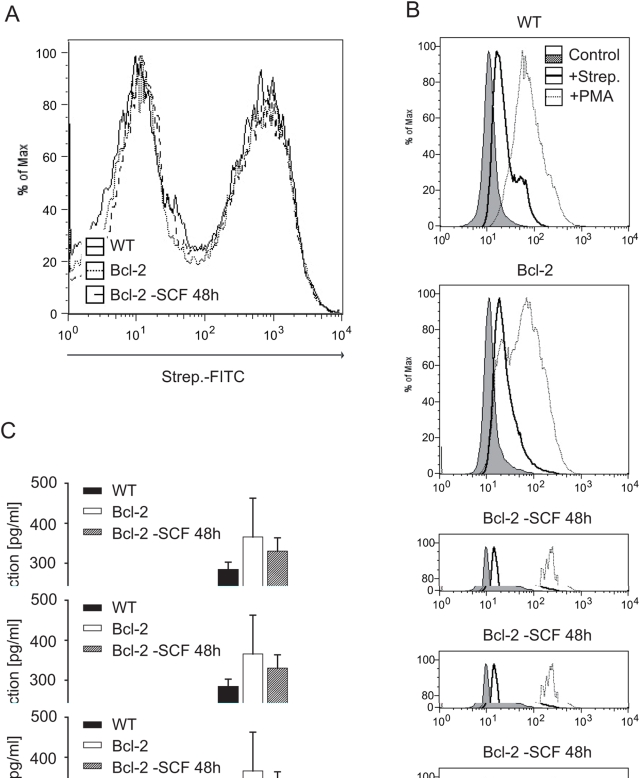
Overexpression of Bcl-2 preserves neutrophil effector functions upon SCF withdrawal. Effector functions of wt or Bcl-2-overexpressing neutrophils differentiated for 5 days were compared with that of Bcl-2 neutrophils differentiated for 5 days and then cultured in the absence of SCF for 48 hours. In all experiments *Streptococcus pneumoniae* and neutrophils were co-incubated at a ratio of 100∶1. (A) Phagocytic uptake of FITC-labelled pneumococci was analysed by flow cytometry after 1 hour co-incubation. Very similar results were seen in 3 independent experiments. (B) Production of reactive oxygen species was measured by the capacity of neutrophils to oxidise dihydrorhodamine. Cells were incubated for one hour with pneumococci (bold line) or with PMA (5 µg/ml, dotted line). Unstimulated cells are shown as shaded histogram. Very similar results were seen in 3 independent experiments. (C) Secretion of IL-1β was measured in supernatants of neutrophils stimulated with pneumococci for 8 hours. Data are mean/SEM of four independent experiments.

We then used this system to address the question whether apoptosis was the pivotal mechanism to shut off neutrophils (as suggested by the *in vivo* results) or whether ageing neutrophils move along a program that disables effector function independently of apoptosis. Neutrophil function was analysed in both wt and Bcl-2-expressing neutrophils immediately upon full differentiation (day 5). In addition, functional parameters were tested in Bcl-2-expressing neutrophils on day 7, following SCF-withdrawal on day 5 (at this time point nearly all wt neutrophils cultured without SCF had died). There was no difference on day 5 between wt and Bcl-2 expressing cells in terms of phagocytosis of FITC-labelled *Streptococcus pneumoniae* (pneumococci, Fig. 4A) or GFP-expressing *E. coli* bacteria (not shown). Exposure of wt and Bcl-2-expressing neutrophils to pneumococci or stimulation with PMA resulted in a very similar level of oxidative burst as measured by the production of oxygen radicals (Fig. 4B). Furthermore, all three groups of cells produced similar levels of IL-1β when stimulated with pneumococci (Fig. 4C) and all thee populations showed similar anti-bacterial activity when co-incubated with pneumococci *in vitro* (not shown). Inhibition of apoptosis by Bcl-2 therefore can maintain function of neutrophils, suggesting that apoptosis is the crucial mechanism to turn off these cells. These data are in accordance with the above *in vivo* results: apoptosis in a pyogenic infection occurs when bacteria have been cleared away (for instance following antibiotic therapy) but if it fails, neutrophils at the site of infection will maintain inflammation and may do tissue damage.

### Treatment with roscovitine induces neutrophil apoptosis and accelerates recovery

In human meningitis, neurological sequelae despite antibiotic treatment are common. Brain tissue damage can also be induced by injection of pneumococcal cell walls, which is reduced when the infiltration of neutrophils is prevented [6]. Neutrophil activity is therefore likely to contribute to disease outcome even in the absence of experimental apoptosis inhibition, and induction of apoptosis subsequent to or accompanying antibiotic therapy may be beneficial for meningitis patients. To test this hypothesis more directly, we used the drug roscovitine to induce apoptosis in neutrophils. Roscovitine is an inhibitor of cyclin-dependent kinases that is in clinical use as an anti-cancer agent. A recent study found that rosocvitine also induces apoptosis in neutrophils, and roscovitine-treatment improved disease in mouse models of sterile inflammation [12]. We found that roscovitine induced apoptosis in neutrophils differentiated from precursors. Apoptosis was implemented by the mitochondrial pathway since it was blocked by Bcl-2 (Fig. S5).

**Figure 5:**
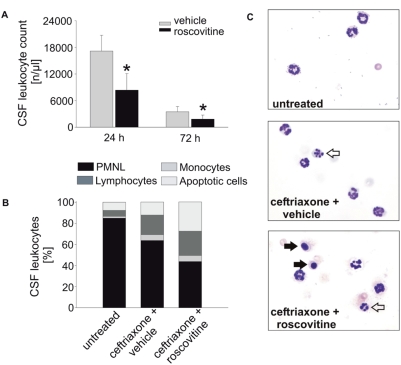
Reduction of CSF leucocyte count and induction of apoptosis by roscovitine *in vivo.* Wild type mice were subjected to pneumococcal meningitis by bacterial injection into the cisterna magna and treated either with a combination of ceftriaxone (100 mg/kg, intraperitoneally) and roscovitine (50 mg/kg intraperitoneally) or ceftriaxone and the vehicle of roscovitine (PBS+20% DMSO). In the case of the 24 h experiments (n = 10 per group), treatment was initiated 18 hours after infection, whereas drugs were given at 24 h and 48 h after infection in the 72 h experiments (n = 11 per group). CSF samples were obtained by puncture of the cisterna magna and analyzed for leucocyte counts (A) as well as the relative proportions of leucocyte subpopulations (B) and apoptotic leucocytes (B,C). (A) Roscovitine treatment significantly reduces CSF leukocyte counts at both observation times. *P<0.05, roscovitine-treated mice compared to vehicle-treated mice. (B,C) CSF samples from untreated, infected mice (n = 6) were used as controls to assess the effect of antibiotic therapy on leucocyte differential counts and apoptosis. (B) Roscovitine therapy increases the proportion of apoptotic leucocytes and decreases the proportion of neutrophils in the CSF. (C) Representative CSF smears of the 24 h time point are shown. Arrows indicate typically apoptotic leucocyte morphology, showing condensed nuclei (filled arrows) and karyorhexis (open arrows).

To test for apoptosis induction by roscovitine and its consequences *in vivo*, we used a different, more virulent strain of *S. pneumoniae* (D39, serogroup 2). In experiments with this strain, 4 out of 11 wt mice and 9 out of 12 Bcl-2-transgenic mice died despite treatment after 24 h (Fig. S6B). This result in wt mice is a good approximation of the lethality of human pneumococcal meningitis. We first tested for the effect of roscovitine on the CSF infiltrate. Wild type mice were infected and treated with antibiotics at 18 h (the earlier treatment was chosen to keep the point of analysis at 24 h; this change also resulted in higher number of mice surviving, see below). One group of mice received roscovitine at the same time point, the other one vehicle (DMSO). As shown in Fig. 5A, roscovitine treatment significantly reduced CSF leucocyte counts as early as 24 h post infection, and a difference was still detectable at 72 h. Analysis of cell composition in CSF in these mice showed enhanced relative numbers of lymphocytes following antibiotic treatment (Fig. 5B). Clearly increased numbers of apoptotic neutrophils, identified by their nuclear pyknosis and karyrhexis were seen in the CSF of roscovitine treated mice (Fig. 5C). Combined treatment with antibiotics and roscovitine thus induces apoptosis *in situ* and very likely by this mechanism reduces the absolute number of neutrophils in the CSF.

**Figure 6:**
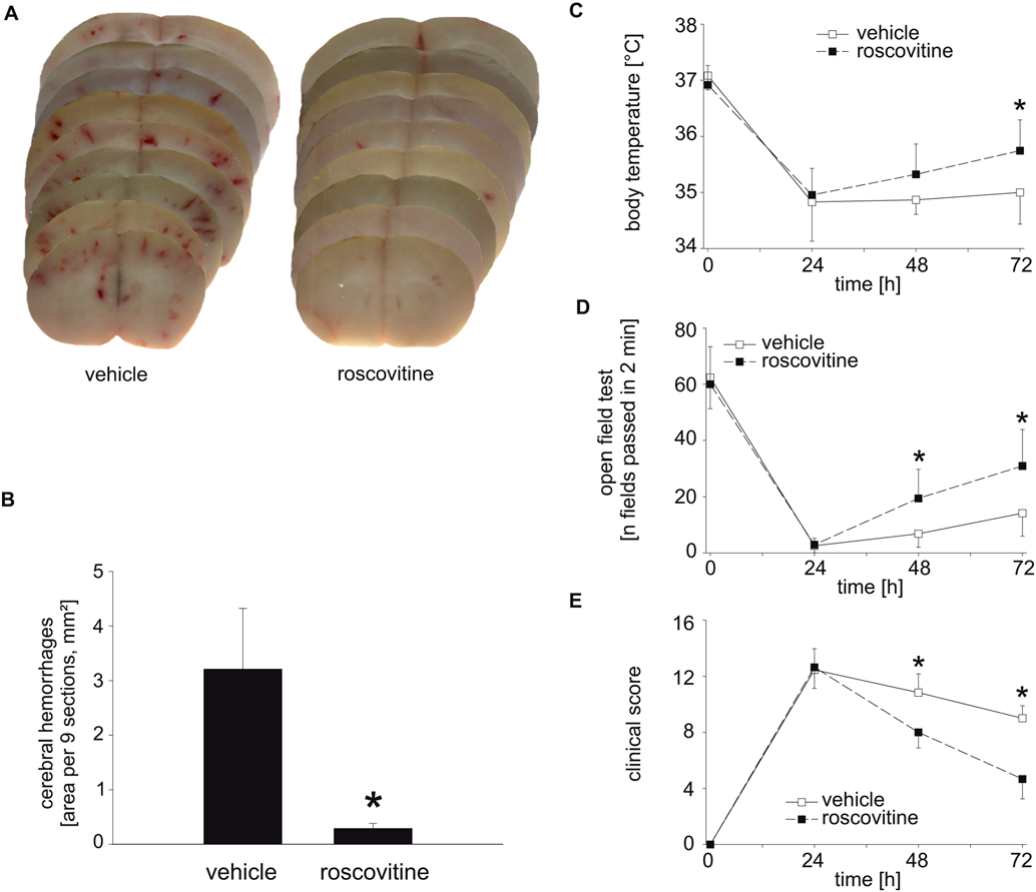
Roscovitine reduces brain tissue damage and accelerates recovery following pneumococcal meningitis. (A) Representative brain sections either obtained from an infected, vehicle-treated or an infected, roscovitine-treated mouse at 72 h after pneumococcal infection are shown. Numerous hemorrhages are predominantly visible in the cortex of vehicle-treated mice. (A,B) Roscovitine is protective against the development of cerebral hemorrhages. (B) The bleeding area was measured using digitalized macroscopic images with the help of ImageTool (UTHSCSA, Texas); *P<0.05, roscovitine-treated mice compared to vehicle-treated mice. The reduction in brain pathology in roscovitine-treated mice was paralleled by a better recovery from the disease, as evidenced by a better reversal of hypothermia (C), a faster recurrence of motor activity (as determined in an open field test (D)), and a more pronounced reduction in the clinical score (the maximum score value is 19 and indicates severe disease and moribund condition whereas a score of 0 is associated with healthy, uninfected mice (E)). *P<0.05, roscovitine-treated mice compared to vehicle-treated mice. (B–E) n = 6 for mice receiving vehicle and n = 9 for roscovitine-treated mice.

When the effect of roscovitine treatment on disease severity was analysed, a profound beneficial effect was seen. Roscovitine reduced number and extent of detectable haemorrhagic events (Fig. 6A,B). Roscovitine significantly improved recovery when mice were compared to groups that had only received antibiotic treatment, as assessed by measuring body temperature (Fig. 6C), physical activity (Fig. 6D) and clinical scores (Fig. 6E). Of the 11 animals treated in each group, 5 died in the group treated with antibiotics plus vehicle while 2 died upon treatment with antibiotics plus roscovitine (Fig. S6C). Roscovitine treatment further led to slightly reduced cerebellar bacterial titres (2.1+/−0.28 vs. 1.74+/−0.69 log cfu/cerebellum; roscovitine/ceftriaxone vs. vehicle/ceftriaxone at 72 h, not significant). Experimental induction of apoptosis in neutrophils can therefore, in combination with antibiotic therapy, correlate with a reduction in tissue damage and disease severity in an acute pyogenic infection.

## Discussion

Our results show that experimental inhibition of apoptosis can exacerbate a pyogenic infection while induction of apoptosis in neutrophils *in vivo* can accelerate recovery from meningitis in mice following antibiotic therapy. Functional *in vitro* analysis further demonstrates that apoptosis is the critical event in terminating neutrophil function, since Bcl-2-overexpressing neutrophils retained an unaltered capacity to phagocytose, to produce oxygen radicals and to secrete IL-1β. These data show that the body's innate immune system has to be tightly controlled to contain inflammation in bacterial infections and identify the apoptosis apparatus as a potential therapeutic target.

Apoptosis is the regular fate of neutrophils and apoptotic neutrophils are disposed of by macrophage-mediated phagocytosis. In neutrophils from Bcl-2-transgenic mice, apoptosis was profoundly inhibited and there was no uptake of Bcl-2-overexpressing, non-apoptotic neutrophils upon prolonged culture. Indeed, neutrophils kept alive by transgenic Bcl-2 maintained their full effector function in all tests we used at a time when almost all wt cells had died. This strongly suggests that, at least *in vitro*, there is no mechanism in place other than apoptosis that would shut off neutrophil effector function; the process of neutrophil ageing is therefore probably identical with the initiation of apoptosis.

It is clear that the end of an immune response has to be tailored to anti-infectious requirements; both too long and too short immune reactions might have negative effects on the organism. In the adaptive immune system, especially in T cells, the termination of the reaction is associated with massive cell death. The primary reason for this cell death may be the reduction of clone size that is necessary because of the massive expansion of T cells at earlier stages of the infection, but it is likely also required to reduce the likelihood of autoimmunity and immunopathology. The experimental reduction of T cell apoptosis, such as in mice deficient for the pro-apoptotic protein Bim can preserve T cell function [23],[24] and causes albeit relatively subtle autoimmunity [25]. In T cells the prevention of hyperinflammation may thus rely not only on apoptosis but also on the necessity of antigen-presence for T cell activity. It is even necessary to retain a certain number of antigen-specific T cells as memory cells, which will resume a state of inactivity but can be quickly re-activated. The situation is different in neutrophils. These cells are programmed to die at the moment of differentiation and although certain inflammatory stimuli, both pathogen and host derived, can prolong the life span of neutrophils [26], they will eventually all die. It is therefore plausible that apoptosis is the only necessary safeguard against neutrophil-mediated hyperinflammation. Circumvention of this safeguard by Bcl-2 over-expression can thus have the drastic effects we have observed. It is not clear whether Bcl-2-transgenic neutrophils actually continuously receive activating stimuli, such as bacterial Pathogen-Associated Molecular Patterns (PAMPS), or whether the initial stimulation is sufficient to set them off on the road to a prolonged inflammation that continues even after the pathogen has gone. If continuous stimulation occurred, the inflammatory activity would have to be below the threshold where new neutrophils are recruited from the blood (otherwise neutrophils would keep coming in from the blood in wt mice), but it is nevertheless possible that levels of inflammatory stimuli are still present that are sufficient for the stimulation of Bcl-2-transgenic neutrophils at the site of infection.

Brain damage despite optimal antibiotic therapy is a severe clinical problem in bacterial meningitis. As shown by earlier work, activated neutrophils can induce tissue damage, and a contribution of neutrophil-mediated inflammation to brain injury during meningitis even in the absence of apoptosis inhibition was likely. It was recently demonstrated that the depletion of neutrophils changes the course of disease in a mouse model of pneumococcal pneumonia [27]. In that model, the depletion of neutrophils prolonged survival of mice and caused differences in histopathological presentation of the lungs, also suggesting that a pro-inflammatory activity of neutrophils contributed to tissue damage. Neutrophils may therefore make regular contributions to disease progression, and this may affect inflammation in tissue-specific ways.

Our data show that roscovitine induces apoptosis in neutrophils *in vivo* and has a beneficial effect on recovery from meningitis. Roscovitine is a clinically used inhibitor of cyclin dependent kinases (cdk). As neutrophils are not cycling, it is surprising that roscovitine induces apoptosis in neutrophils, and this may be mediated by other, unknown cellular targets of the drug (or cdks may fulfil other functions in neutrophils). Apoptosis was blocked by Bcl-2, demonstrating the involvement of the mitochondrial apoptosis pathway. In clinical use, roscovitine does not have major side effects, and its use as an anti-inflammatory drug may therefore be considered. Most importantly, induction of apoptosis could improve the outcome of the acute infection, and this strategy could be important in human inflammatory diseases. As neutrophil apoptosis can also be prevented by cytokines and by bacterial components, therapeutic targeting of such stimuli might also be worth considering.

Apoptotic neutrophil death *in situ* thus has two anti-inflammatory effects that may be of importance. The obvious, direct effect is that the cells die and thus stop producing pro-inflammatory mediators. The second, indirect effect is that the dampening activity of macrophages that normally results from the uptake of apoptotic neutrophils is absent. The reduced levels of TGF-β in the brains of Bcl-2 transgenic mice observed here support this interpretation.

In conclusion, our data show that inhibition of apoptosis can preserve neutrophil function, and that this inhibition can promote prolonged, tissue-damaging inflammation in a pyogenic infection. Factors affecting the lifespan of neutrophils in inflamed tissue are therefore promising targets in anti-inflammatory therapy. It could be speculated that besides the therapeutic induction of apoptosis in neutrophils to reduce inflammation, approaches aiming at increased survival of neutrophils may be worth considering in situations where neutrophil function is compromised.

## Materials and Methods

### Ethics statement

All animal experiments had been approved by the Regierung von Oberbayern (Government of Upper Bavaria).

### Cell lines and bacteria

Hoxb8 neutrophil progenitors derived from WT and *bcl2*-transgenic mice as described in [22] were cultured in Optimem medium (Invitrogen, Karlsruhe, Germany) supplemented with 10% FCS (PAA, Cölbe, Germany), 30 µM β-mercaptoethanol (Sigma, Munich, Germany), antibiotics (100 IU/ml penicillin G and 100 IU/ml streptomycin sulfate, Biochrom), 4% supernatant from stem cell factor (SCF)-producing Chinese Hamster Ovarian cells, and 1 µM oestrogen (Sigma). The retroviral expression vectors for HoxB8 and SCF-producing cells were provided my Dr. Mark Kamps, San Diego. Neutrophil differentiation was induced by removal of oestrogen, and subsequent culture for 5 days in medium containing 2% SCF. Murine RAW 264.7 macrophages were cultured in Very-Low-Endotoxin RPMI 1640 (Biochrom, Berlin, Germany) supplemented with 10% FCS and antibiotics. *Streptococcus pneumoniae* serotype 3 bacteria were grown in Trypticase soy yeast extract medium (3% trypticase soy broth, 0.3% yeast extract) overnight at 37°C. To assess differentiation status, cells were subjected to cytospin and Giemsa stain or stained with rabbit anti-Gr1-FITC (1:100, BD Pharmingen, Heidelberg, Germany) in PBS/0.5% BSA for 30 minutes prior to flow cytometry analysis with a FACS Calibur (Beckton Dickinson, Heidelberg, Germany).

### Apoptosis assays and uptake of apoptotic neutrophils

For analysis of apoptosis by AnnexinV-PI staining, Hoxb8 neutrophils were differentiated for 5 days, then washed in PBS/10% FCS with or without SCF (2%), and cultured in the presence or absence of SCF (2%) for 24 or 48 hours. For induction of apoptosis by Roscovitin, differentiated neutrophils washed in the presence of SCF were cultured for 8 or 24 hours with SCF (2%) plus 25 or 50 µM Roscovitin (Calbiochem). After washing with AnnexinV-binding buffer (10 mM Hepes, 140 mM NaCl, 2.5 mM CaCl_2_), cells were incubated with AnnexinV-FITC (1:50, BD Pharmingen) and propidium iodide (1:500, Sigma) for 20 minutes, followed by flow cytometry analysis. For analysis of caspase-3 activity, neutrophils differentiated for 5 days and washed as described above were cultured with or without SCF (2%) for 8 or 48 hours. Cells were washed with PBS, fixed in 3.7% formaldehyde and permeabilised with 1% Saponin (Sigma). Staining was performed with rabbit anti-active caspase-3 (Abcam, Cambridge, UK) in PBS/0.5% BSA for 40 minutes, followed by secondary donkey anti-rabbit-FITC (Dianova, Hamburg, Germany) for 30 minutes. Neutrophils were analysed by flow cytometry.

RAW macrophages were stained red with PKH26 according to the manufacturer's instructions (Sigma) and seeded overnight into 24-well plates at 2×10^5^/well. Neutrophils differentiated for 5 days were deprived of SCF as described above and cultured for 16, 48, or 72 hours. Immediately prior to the phagocytosis assay, part of the neutrophils were stained for AnnexinV-PI as described, while the remainder was stained green with CSFE (1 µM, Invitrogen) at 37°C for 10 minutes, followed by quenching of unreacted dye in Optimem medium for 30 minutes. Stained neutrophils were resuspended in RPMI medium and added to macrophage monolayers at 1×10^6^/well. After 4 hours, cells were detached with accutase (PAA) and subjected to flow cytometry analysis.

### Analysis of neutrophil effector function

Hoxb8 neutrophils were differentiated for 5 days and deprived of SCF for 48 hours, as described for the apoptosis assays. For analysis of phagocytic capacity, pneumococci grown overnight were first washed, stained with 20 µg/ml FITC (Sigma) in carbonate buffer (0.1 M Na_2_CO_3_, pH 9.2) for 1 hour at 37°C, and the optical density of the suspension at 600 nm was adjusted to 1.0 after washes with PBS. Four times 10^5^ neutrophils were co-incubated with pneumococci at a bacteria-to-cell ratio of 100∶1 for 1 hour at 37°C while shaking, followed by flow cytometry analysis. In order to analyse the production of reactive oxygen species, 4×10^5^ neutrophils were co-incubated with pneumococci at a bacteria-to-cell ratio of 100∶1 at 37°C while shaking. After 30 minutes, 2 µM dihydrorhodamine (Sigma) was added and co-incubation was continued for another 30 minutes. Cells were analysed by flow cytometry. For the measurement of IL-1β production, 5×10^5^ neutrophils were co-incubated with pneumococci at a bacteria-to-cell ratio of 100∶1 in 96-well plates for 8 hours. IL-1β was quantified in supernatants using the Mouse IL-1β DuoSet ELISA kit (R&D Systems, Wiesbaden, Germany) according to the manufacturer's instructions.

### Mouse meningitis model

The model used in this study has been described previously [15],[21]. Briefly, male C67BL/6 mice were weighed and clinically examined. Meningitis was induced by transcutaneous injection of 15 µl of a bacterial suspension containing 10^7^ colony forming units (cfu) per ml of *Streptococcus pneumoniae* type 3 into the cisterna magna under short-term anesthesia with halothane. Twenty-four hours after infection, mice were evaluated clinically and treated with ceftriaxone (100 mg/kg intraperitoneally). After 48 hours (72 hours after infection), mice were again clinically evaluated, reweighed, and the body temperature was measured via a rectal probe. Then, mice were anaesthetized with 100 mg/kg ketamine and 5 mg/kg xylazine. A catheter was inserted into the cisterna magna to determine CSF leukocyte counts, as well as relative proportions of leucocyte subpopulations and apoptotic leucocytes. Subsequently, blood samples were taken by transcardial puncture. After deep anesthesia with ketamine, mice were perfused transcardially with 15 ml of ice-cold phosphate-buffered saline (PBS) containing 10 U/ml heparin. The brains were removed and rapidly frozen. In a subset of experiments, mice were processed in the same way at 24 or 48 hours after infection (time point of antibiotic therapy in the other groups).

### Bacterial titers

Cerebella were dissected and homogenized in sterile saline. Homogenates were diluted serially in sterile saline, plated on blood agar plates, and cultured for 24 h at 37°C with 5% CO_2_.

### Determination of the blood–brain barrier integrity

Mouse brain homogenates were examined for diffusion of albumin using ELISA as described previously [28].

### Analysis of cerebral bleeding

Mice brains were cut in a frontal plane into 10 µm thick sections. Beginning from the anterior parts of the lateral ventricles, 9 serial sections were photographed with a digital camera in 0.3 mm intervals throughout the ventricle system. The areas of the lateral ventricles and the third ventricle were measured (Image tool, UTHSCSA, Texas, USA) and the volume of the ventricle size was estimated (Σ ventricle area/9 pictures×0.3 mm). Haemorrhagic spots were counted and the bleeding area was measured (Image tool, UTHSCSA, Texas, USA). Cryosections of mice brains were H&E stained. In addition, selected brains were formaline fixed, paraffin embedded, and sections were stained with H&E.

### Immunohistochemical detection of neutrophils

Ten-µm-thick coronal brain sections containing the lateral ventricles and hippocampal tissue were stained with a rat anti-mouse GR-1 monoclonal antibody (RB6-8C5; BD Biosciences, Erembodegem, Belgium) for evaluation of brain neutrophil infiltration. After quenching endogenous peroxidase activity with 0.3% methanolic hydrogen peroxide and blockage of non-specific binding by 10% normal rabbit serum, brain sections were incubated with the anti-GR-1 antibody diluted 1∶10 overnight at 4°C. Labelled cells were visualized using biotinylated rabbit anti-rat IgG at a 1∶200 dilution, followed by horseradish peroxidase-conjugated streptavidin and then 3,3-diaminobenzidine as a chromogen (all from Vector Laboratories, Burlingame, USA). After counterstaining with Mayer's hematoxylin solution, tissue sections were imaged at a magnification of 35 using a digital video camera connected to a PC.

### Immunoassays for pro- and anti- inflammatory mediators

IL-1β, G-CSF, IL-6, IL-1 receptor antagonist and immunoreactive TGF-ß were determined using commercially available ELISA kits (Quantikine Assay kits, R&D Systems GmbH, Wiesbaden-Nordenstadt, FRG). Frozen brain sections (with a total thickness of 1.8 mm) were homogenized in lysis buffer, then centrifuged at 12,000 rpm for 15 min at 4°C, and 50 µl of the supernatant was used for each determination. Additionally, the protein concentration of the supernatant was measured using the Nanoquant assay (Carl Roth GmbH, Karlsruhe, FRG) and cytokine concentrations are expressed with reference to total protein concentration.

### Depletion of neutrophils

Wt or Bcl-2 transgenic mice were injected intraperitoneally with 250 µg rat anti-mouse GR-1 monoclonal antibody 6 hours before infection as reported previously [29]. The control group of mice (n = 4) received intraperitoneal injection of 250 µg purified rat IgG_2b_ isotype control antibody. Mice were infected and analyzed as above.

### Statistical analysis

The principal statistical test used for comparison of multiple groups (for example, anti-GR-1-treated Bcl-2 transgenic and wild type mice as well as their respective isotype antibody-treated controls) was one-way analysis of variance and Scheffe's test. In order to test statistical significance between two groups (for example, roscovitine- and vehicle-treated wild type mice), we used unpaired Student's t test. The statistical difference of mortality was determined by the Kaplan-Meyer log-rank test. Differences were considered significant at *p*<0.05. Data are expressed as mean ± SD.

## Supporting Information

Figure S1 Continued presence of Bcl-2 transgenic neutrophils in the CNS following pneumococcal infection. (A) H&E stained sections at 72 h of pneumococcal infection show continued leucocyte infiltration of the lateral ventricle (open arrow) in infected Bcl-2 transgenic mice but few cells in the same area in wt mice. (B) Staining for neutrophils by anti-Gr-1 immunohistochemistry at 72 h post infection shows neutrophil infiltration of the subarachnoidal space (left, open arrow) and neutrophils in the proximity of microvessels (right, closed arrow). Very few neutrophils were seen in wt mice in similar sections at this time point (not shown).
(0.10 MB PDF)

Figure S2 Neutrophil depletion reduces CSF inflammation and brain tissue injury in wt and Bcl-2 transgenic mice. Bcl-2 transgenic mice (n = 5) and wild type mice (n = 5) were rendered neutropenic by intraperitoneal injection of 250 µg rat anti-mouse GR-1 monoclonal antibody 6 hours before infection. The control Bcl-2 transgenic and wild type mice (n = 5 per group) received intraperitoneal injection of 250 µg purified rat IgG2b isotype control antibody. Mice were treated with 100 µg/kg ceftriaxone 18 hours after infection and analyzed 24 hours later. (A) To verify neutrophil depletion, blood samples were obtained at the time of sacrifice by cardiac puncture. The total leukocyte count was determined using blood samples diluted in Turk's solution counted in a Neubauer chamber, and differential leukocyte counts were performed on thin blood smears stained by the May-Gruenwald-Giemsa method. Anti-Gr-1 treatment resulted in a 93.2% and 92.6% reduction in mean neutrophil counts in Bcl-2 transgenic and wild type mice compared with the isotype controls. (B,C) CSF samples were obtained by puncture of the cisterna magna and analyzed for leucocyte counts (B) as well as the relative proportions of leucocyte subpopulations and apoptotic leucocytes (C). (B) CSF leukocyte counts were 84.1 and 78.6% lower in anti-GR1-treated Bcl-2 transgenic and wild type mice compared to the respective isotype controls. (C) Differential leukocyte counts revealed neutrophils as the predominant leukocyte subpopulation at this disease stage. Overexpression of Bcl-2 in hematopoietic cells decreased the proportion of apoptotic leucocytes and increased the proportion of neutrophils in the CSF. (D) Bacterial titres were determined in cerebellar homogenates serially diluted in sterile saline and plated on blood agar plates. Bacterial killing in the CNS was not affected by neutrophil depletion. (E) Representative brain sections either obtained from an isotype- or anti-Gr-1 treated Bcl-2 transgenic animal at 42 hours after pneumococcal infection are shown. Cerebral hemorrhages are clearly visible in the cortex of the isotype-treated mouse. (F) Hemorrhagic spots were counted using digitalized macroscopic images using ImageTool (UTHSCSA, Texas). * P<0.05, compared to isotype-injected mice using one-way analysis of variance and Scheffe's test.
(0.03 MB PDF)

Figure S3 Differentiation of wt and Bcl-2-expressing neutrophils from progenitor lines *in vitro*. Top, wt or Bcl-2-overexpressing progenitors were cultured in the presence or absence of oestrogen in the presence of SCF for 5 days. Cells were subjected to Giemsa stain to reveal their nuclear morphology. Note the typical segmentation and doughnut shape of mouse neutrophils, Bottom, differentiation of wt or Bcl-2-transgenic progenitors was induced by oestrogen withdrawal. Surface expression of the neutrophil marker Gr-1 was measured by flow cytometry on days 1, 3, and 5. The reduction in cell size at the later stage of differentiation may contribute to the slight reduction in Gr-1 expression after day 3.
(0.12 MB PDF)

Figure S4 Inhibition of cell death, caspase-activation and macrophage uptake in neutrophils by Bcl-2 *in vitro* (A), wt or Bcl-2-overexpressing neutrophils differentiated for 5 days were in addition cultured for 24 or 48 hours in the presence or absence of SCF. Cells were then stained with AnnexinV-FITC and propidium iodide (PI) and analysed by flow cytometry. Dot blots show staining of wt (top) or Bcl-2-transgenic (bottom) cells that were cultured in the presence (left) or absence (right) of SCF. (B) Neutrophils differentiated for 5 days were cultured for 8 or 48 hours in the presence or absence of SCF, followed by staining for active caspase-3. (C) Neutrophils differentiated for 5 days were cultured in the absence of SCF for the indicated periods of time, and stained with AnnexinV-PI (upper panel) or CFSE. Green-fluorescent (CFSE-stained) neutrophils were then added to cultures of RAW macrophages stained with the red dye PKH26 (ratio neutrophil∶macrophages, 5∶1). After 4 h of co-incubation, cultures were subjected to flow cytometric analysis. The phagocytic (green-fluorescent) macrophage population is found in the upper right gate (lower panel).
(0.26 MB PDF)

Figure S5 Roscovitine induces Bcl-2-inhibitable apoptosis in neutrophils *in vitro*. Wt or Bcl-2-overexpressing neutrophils differentiated for 5 days were cultured for 8 or 24 hours in the presence of SCF and vehicle (DMSO) or 25 or 50 µM roscovitine (hatched bars). Cells were then stained with AnnexinV-FITC and propidium iodide (PI) and analysed by flow cytometry. Graphs show the percentage of AnnexinV-PI double-positive cells. Bars/error bars indicate mean/SEM of three independent experiments.
(0.01 MB PDF)

Figure S6 Survival curves for wild type and Bcl-2 transgenic mice after intracisternal inoculation with serotype 3 or serotype 2 pneumococci. Death due to meningitis generally occurred within 8 hours after the initiation of antibiotic therapy (which was started 24 hours after infection). (A) Meningitis induced by a serotype 3 pneumococcus (clinical isolate) resulted in a relatively moderate disease course, as indicated by zero-mortality in wild type mice (wt). Infection of Bcl-2 transgenic mice (Bcl2) with this strain resulted in death of 3 of 12 mice. (B) Compared to the serotype 3 strain, infection of wt mice with the D39 strain led to a significantly higher mortality rate of 36% (p = 0.019). In Bcl-2 transgenic mice, the D39 strain caused death in 9 of 12 animals (p = 0.082, not significant, compared to wt). (C) Adjuvant therapy with roscovitine tended to reduce the mortality rate of wt mice infected with the D39 strain (p = 0.281, not significant, compared to vehicle-treated wt). The statistical difference of mortality was determined by the Kaplan-Meyer log-rank test.
(0.02 MB PDF)
