# Supplementary figures and images for: Apoptosis Is Essential for Neutrophil Functional Shutdown and Determines Tissue Damage in Experimental Pneumococcal Meningitis
Source: PLoS Pathog. 2009 May 29;5(5):e1000461. doi: 10.1371/journal.ppat.1000461 (PMC2682662; doi:10.1371/journal.ppat.1000461)

**Supplemental figure S1**

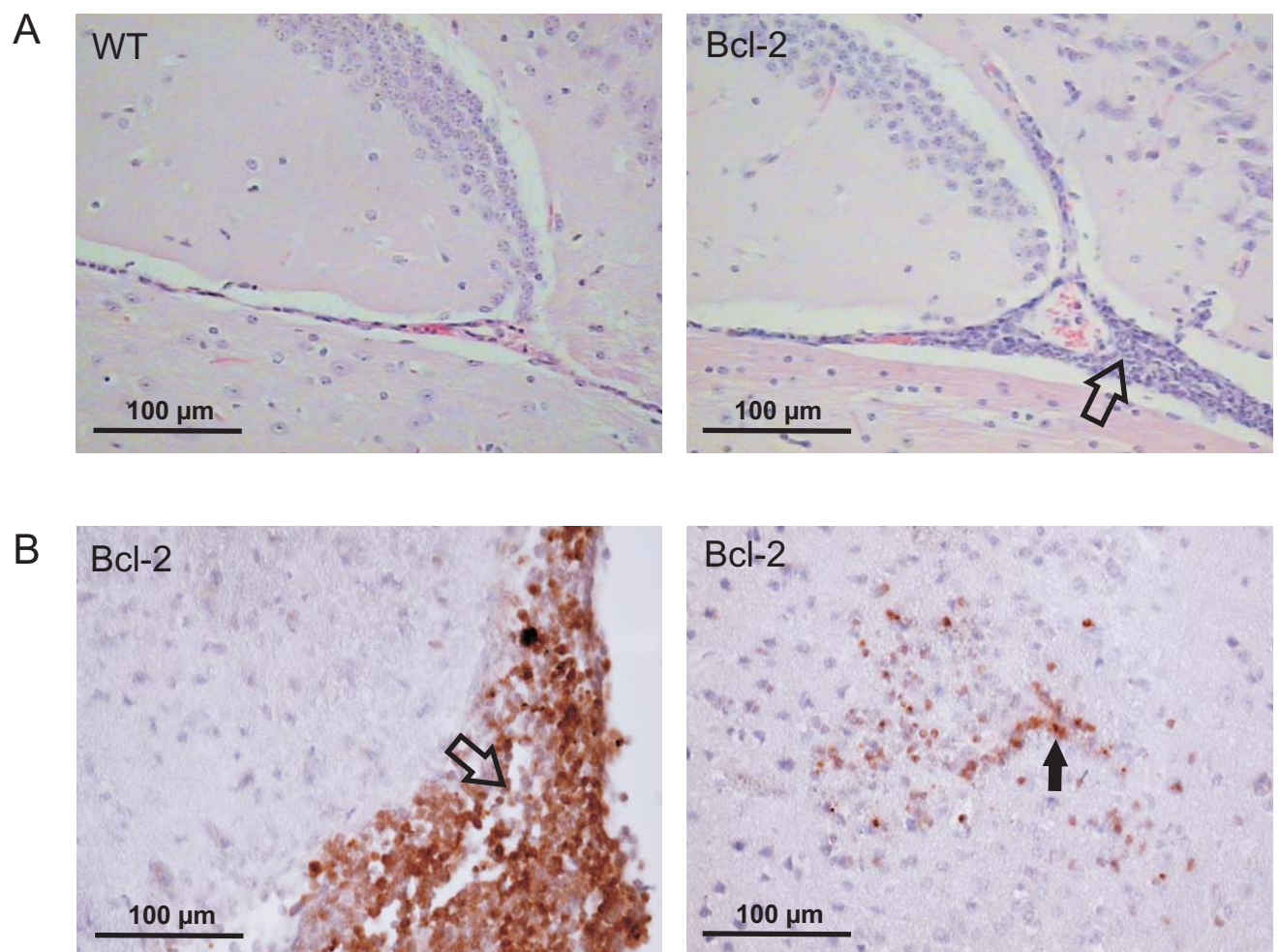

Supplement: Figure S1 — Continued presence of Bcl-2 transgenic neutrophils in the CNS following pneumococcal infection. (A) H&E stained sections at 72 h of pneumococcal infection show continued leucocyte infiltration of the lateral ventricle (open arrow) in infected Bcl-2 transgenic mice but few cells in the same area in wt mice. (B) Staining for neutrophils by anti-Gr-1 immunohistochemistry at 72 h post infection shows neutrophil infiltration of the subarachnoidal space (left, open arrow) and neutrophils in the proximity of microvessels (right, closed arrow). Very few neutrophils were seen in wt mice in similar sections at this time point (not shown). (0.10 MB PDF) [file ppat.1000461.s001.pdf]

Supplemental Figure S2

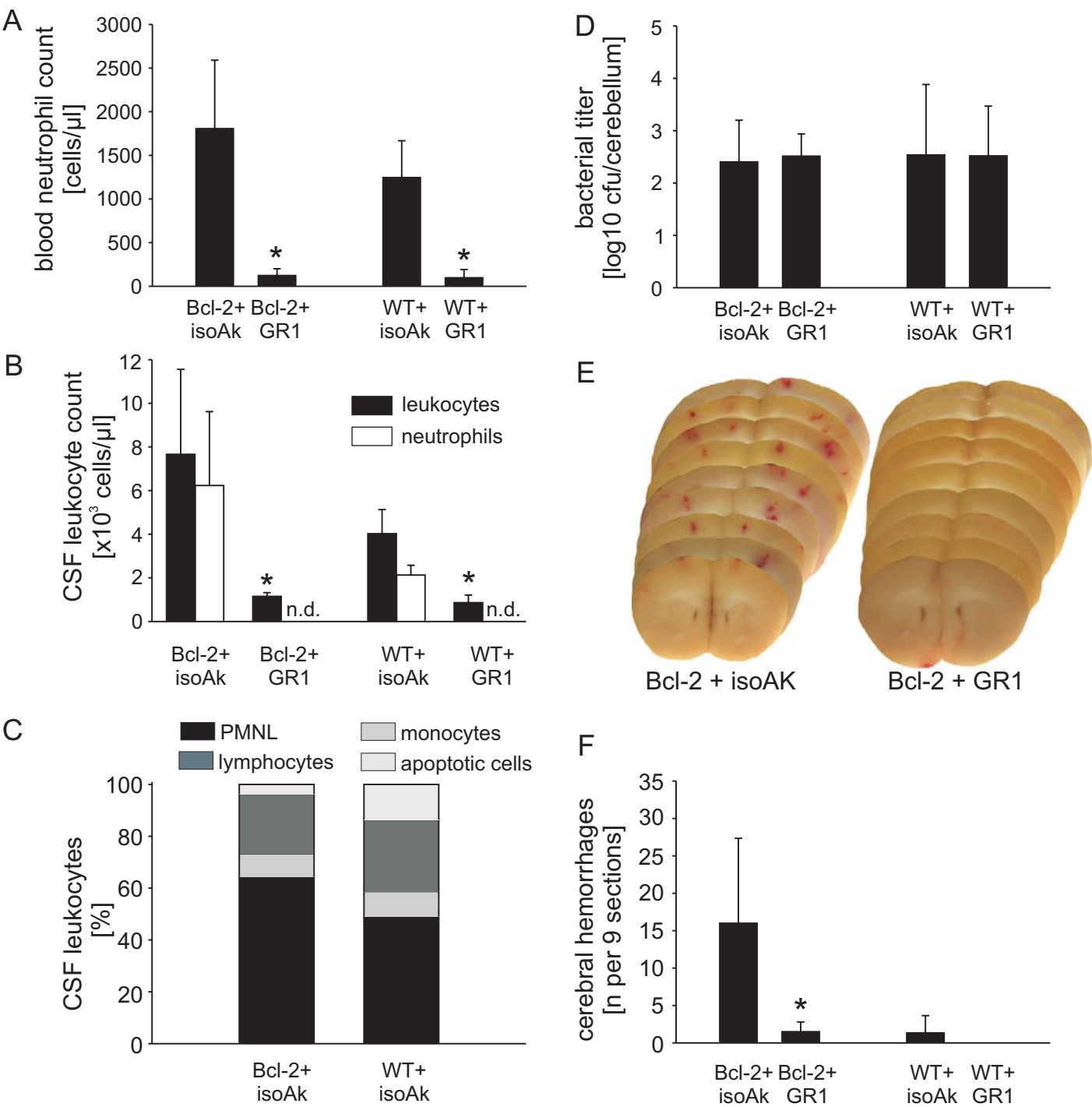

Supplement: Figure S2 — Neutrophil depletion reduces CSF inflammation and brain tissue injury in wt and Bcl-2 transgenic mice. Bcl-2 transgenic mice (n = 5) and wild type mice (n = 5) were rendered neutropenic by intraperitoneal injection of 250 µg rat anti-mouse GR-1 monoclonal antibody 6 hours before infection. The control Bcl-2 transgenic and wild type mice (n = 5 per group) received intraperitoneal injection of 250 µg purified rat IgG2b isotype control antibody. Mice were treated with 100 µg/kg ceftriaxone 18 hours after infection and analyzed 24 hours later. (A) To verify neutrophil depletion, blood samples were obtained at the time of sacrifice by cardiac puncture. The total leukocyte count was determined using blood samples diluted in Turk's solution counted in a Neubauer chamber, and differential leukocyte counts were performed on thin blood smears stained by the May-Gruenwald-Giemsa method. Anti-Gr-1 treatment resulted in a 93.2% and 92.6% reduction in mean neutrophil counts in Bcl-2 transgenic and wild type mice compared with the isotype controls. (B,C) CSF samples were obtained by puncture of the cisterna magna and analyzed for leucocyte counts (B) as well as the relative proportions of leucocyte subpopulations and apoptotic leucocytes (C). (B) CSF leukocyte counts were 84.1 and 78.6% lower in anti-GR1-treated Bcl-2 transgenic and wild type mice compared to the respective isotype controls. (C) Differential leukocyte counts revealed neutrophils as the predominant leukocyte subpopulation at this disease stage. Overexpression of Bcl-2 in hematopoietic cells decreased the proportion of apoptotic leucocytes and increased the proportion of neutrophils in the CSF. (D) Bacterial titres were determined in cerebellar homogenates serially diluted in sterile saline and plated on blood agar plates. Bacterial killing in the CNS was not affected by neutrophil depletion. (E) Representative brain sections either obtained from an isotype- or anti-Gr-1 treated Bcl-2 transgenic animal at 42 hours a [file ppat.1000461.s002.pdf]

Supplemental figure S3

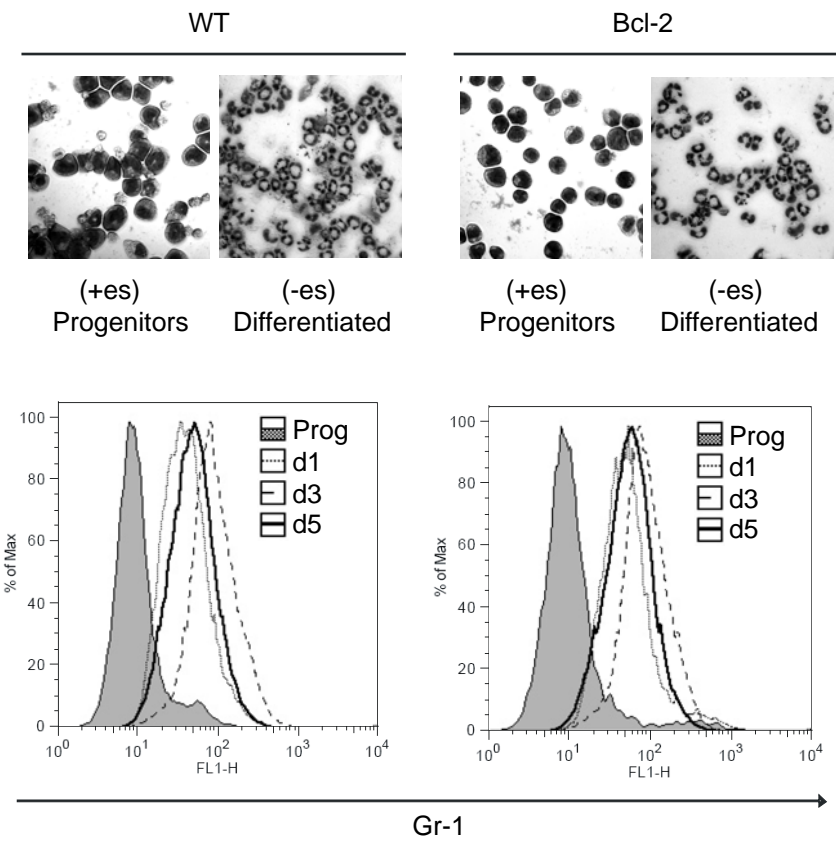

Supplement: Figure S3 — Differentiation of wt and Bcl-2-expressing neutrophils from progenitor lines in vitro. Top, wt or Bcl-2-overexpressing progenitors were cultured in the presence or absence of oestrogen in the presence of SCF for 5 days. Cells were subjected to Giemsa stain to reveal their nuclear morphology. Note the typical segmentation and doughnut shape of mouse neutrophils, Bottom, differentiation of wt or Bcl-2-transgenic progenitors was induced by oestrogen withdrawal. Surface expression of the neutrophil marker Gr-1 was measured by flow cytometry on days 1, 3, and 5. The reduction in cell size at the later stage of differentiation may contribute to the slight reduction in Gr-1 expression after day 3. (0.12 MB PDF) [file ppat.1000461.s003.pdf]

Supplemental figure S4

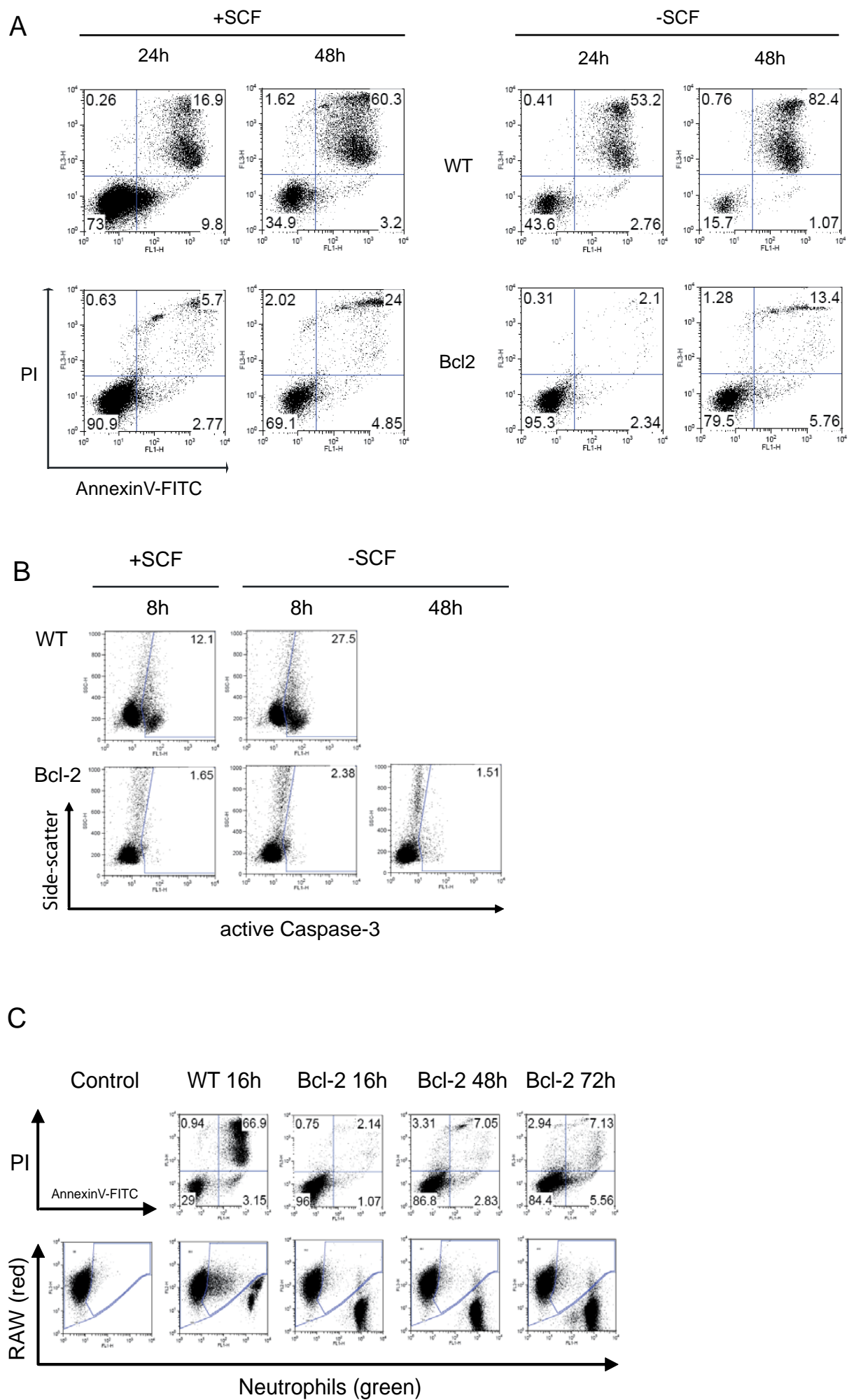

Supplement: Figure S4 — Inhibition of cell death, caspase-activation and macrophage uptake in neutrophils by Bcl-2 in vitro (A), wt or Bcl-2-overexpressing neutrophils differentiated for 5 days were in addition cultured for 24 or 48 hours in the presence or absence of SCF. Cells were then stained with AnnexinV-FITC and propidium iodide (PI) and analysed by flow cytometry. Dot blots show staining of wt (top) or Bcl-2-transgenic (bottom) cells that were cultured in the presence (left) or absence (right) of SCF. (B) Neutrophils differentiated for 5 days were cultured for 8 or 48 hours in the presence or absence of SCF, followed by staining for active caspase-3. (C) Neutrophils differentiated for 5 days were cultured in the absence of SCF for the indicated periods of time, and stained with AnnexinV-PI (upper panel) or CFSE. Green-fluorescent (CFSE-stained) neutrophils were then added to cultures of RAW macrophages stained with the red dye PKH26 (ratio neutrophil∶macrophages, 5∶1). After 4 h of co-incubation, cultures were subjected to flow cytometric analysis. The phagocytic (green-fluorescent) macrophage population is found in the upper right gate (lower panel). (0.26 MB PDF) [file ppat.1000461.s004.pdf]

Supplemental figure S5

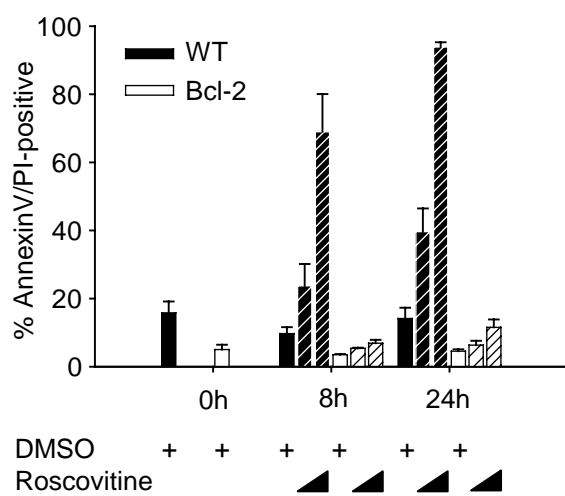

Supplement: Figure S5 — Roscovitine induces Bcl-2-inhibitable apoptosis in neutrophils in vitro. Wt or Bcl-2-overexpressing neutrophils differentiated for 5 days were cultured for 8 or 24 hours in the presence of SCF and vehicle (DMSO) or 25 or 50 µM roscovitine (hatched bars). Cells were then stained with AnnexinV-FITC and propidium iodide (PI) and analysed by flow cytometry. Graphs show the percentage of AnnexinV-PI double-positive cells. Bars/error bars indicate mean/SEM of three independent experiments. (0.01 MB PDF) [file ppat.1000461.s005.pdf]

Supplemental Figure S6

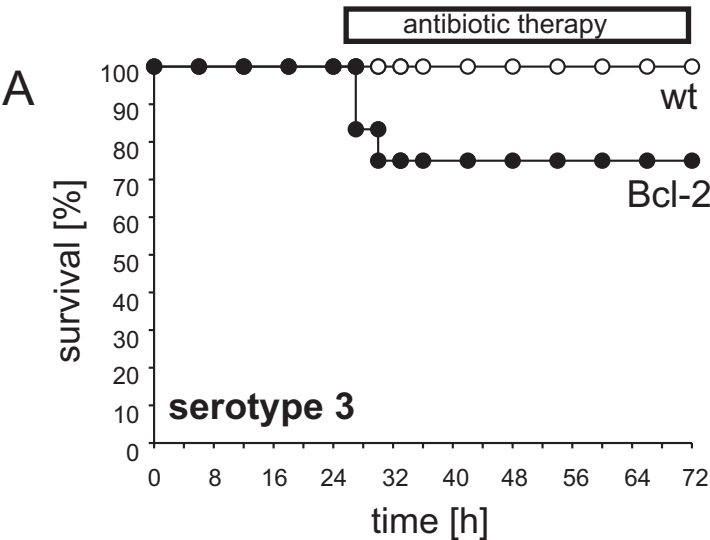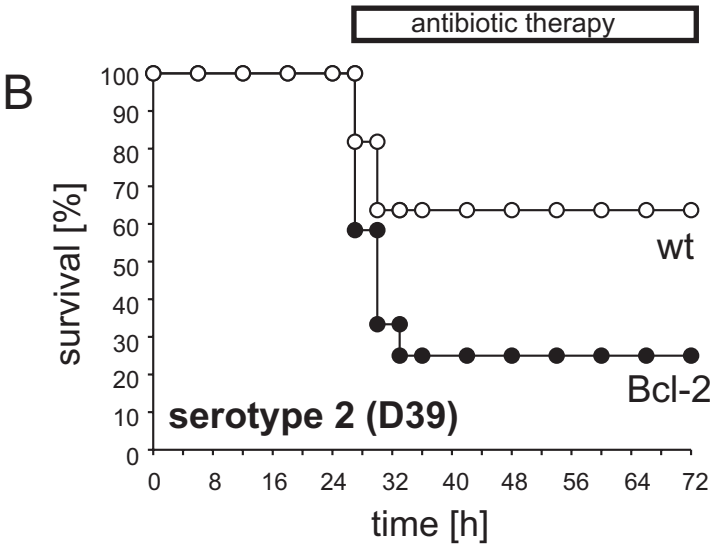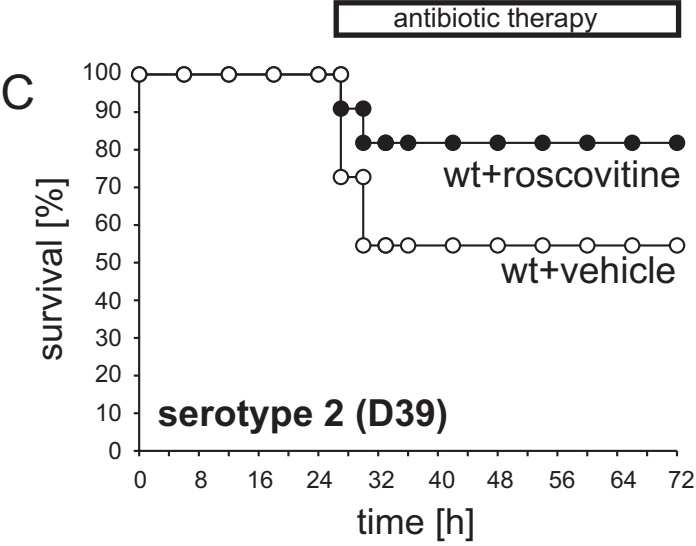

Supplement: Figure S6 — Survival curves for wild type and Bcl-2 transgenic mice after intracisternal inoculation with serotype 3 or serotype 2 pneumococci. Death due to meningitis generally occurred within 8 hours after the initiation of antibiotic therapy (which was started 24 hours after infection). (A) Meningitis induced by a serotype 3 pneumococcus (clinical isolate) resulted in a relatively moderate disease course, as indicated by zero-mortality in wild type mice (wt). Infection of Bcl-2 transgenic mice (Bcl2) with this strain resulted in death of 3 of 12 mice. (B) Compared to the serotype 3 strain, infection of wt mice with the D39 strain led to a significantly higher mortality rate of 36% (p = 0.019). In Bcl-2 transgenic mice, the D39 strain caused death in 9 of 12 animals (p = 0.082, not significant, compared to wt). (C) Adjuvant therapy with roscovitine tended to reduce the mortality rate of wt mice infected with the D39 strain (p = 0.281, not significant, compared to vehicle-treated wt). The statistical difference of mortality was determined by the Kaplan-Meyer log-rank test. (0.02 MB PDF) [file ppat.1000461.s006.pdf]
